# Supplementary material for: Resolving the full spectrum of human genome variation using Linked-Reads
Source: Genome Res. 2019 Apr;29(4):635–45. doi: 10.1101/gr.234443.118 (PMC6442396; doi:10.1101/gr.234443.118)
Supplement: Supplemental Material [file supp_gr.234443.118_Supplemental_longranger-2.2.zip › longranger-2.2.tar.bz2!/lr2.2-public/lib/stan/cmdstan/stan/doc/api/html/diag__post__multiply_8hpp_source.html]

Stan: src/stan/math/matrix/diag\_post\_multiply.hpp Source File


|  |  |
| --- | --- |
|  | Stan  2.5.0  probability, sampling & optimization |


- Main Page
- Related Pages
- Namespaces
- Classes
- Files

- File List
- File Members

All Classes Namespaces Files Functions Variables Typedefs Enumerations Enumerator Friends Macros Pages

- src
- stan
- math
- matrix

diag\_post\_multiply.hpp

Go to the documentation of this file.

1 #ifndef STAN\_\_MATH\_\_MATRIX\_\_DIAG\_POST\_MULTIPLY\_HPP

2 #define STAN\_\_MATH\_\_MATRIX\_\_DIAG\_POST\_MULTIPLY\_HPP

3

4 #include <stdexcept>

5 #include <boost/math/tools/promotion.hpp>

6 #include <stan/math/matrix/Eigen.hpp>

7

8 namespace stan {

9  namespace math {

10

11  template <typename T1, typename T2, int R1,int C1,int R2,int C2>

12  Eigen::Matrix<typename boost::math::tools::promote\_args<T1,T2>::type, R1, C1>

13  diag\_post\_multiply(const Eigen::Matrix<T1,R1,C1>& m1,

14  const Eigen::Matrix<T2,R2,C2>& m2) {

15  if (m2.cols() != 1 && m2.rows() != 1)

16  throw std::domain\_error("m2 must be a vector");

17  int m1\_cols = m1.cols();

18  if (m2.size() != m1\_cols)

19  throw std::domain\_error("m2 must have same length as m1 has columns");

20  int m1\_rows = m1.rows();

21  Eigen::Matrix<typename boost::math::tools::promote\_args<T1,T2>::type, R1, C1>

22  result(m1\_rows, m1\_cols);

23

24  for (int j = 0; j < m1\_cols; ++j)

25  for (int i = 0; i < m1\_rows; ++i)

26  result(i,j) = m2(j) \* m1(i,j);

27  return result;

28  }

29

30  }

31 }

32 #endif

stan::math::diag\_post\_multiply

Eigen::Matrix< typename boost::math::tools::promote\_args< T1, T2 >::type, R1, C1 > diag\_post\_multiply(const Eigen::Matrix< T1, R1, C1 > &m1, const Eigen::Matrix< T2, R2, C2 > &m2)

**Definition:** diag\_post\_multiply.hpp:13

Eigen.hpp


---

[ Stan Home Page ]

*© 2011–2014,
Stan Development Team.*
